# Supplementary material for: Vitamin B2 enables regulation of fasting glucose availability
Source: eLife. 2023 Jul 7;12:e84077. doi: 10.7554/eLife.84077 (PMC10328530; doi:10.7554/eLife.84077)
Supplement: Figure 2—source data 2. — Data are mean ± SEM. The number of animals in each group is indicated in parentheses. Statistical significance (p) was determined by Mann-Whitney tests. [file elife-84077-fig2-data2.docx]

|  | **Cholesterol** | **TGs** | **Lactate** | **FFA** | **b-HB** | **Glycerol** |
| --- | --- | --- | --- | --- | --- | --- |
|  | **(mg/dl)** | **(mg/dl)** | **(mM)** | **(μM)** | **(mM)** | **(μM)** |
| 99% Ctrl | 146.5 ± 7.2 (5) | 100.9 ± 6.7 (5) | 4.5 ± 0.5 (5) | 994.1 ± 84.9 (5) | 0.19 ± 0.02 (5) | 105.4 ± 15.4 (5) |
| 99% B2D | 134.3 ± 11.0 (4) | 126.6 ± 17.7 (4) | 6.4 ± 0.4 (4) | 1128.5 ± 39.0 (5) | 0.27 ± 0.03 (5) | 134.0 ± 13.3 (4) |
| p | 0.36 | 0.18 | 0.18 | 0.15 | 0.08 | 0.22 |
| Ctrl + FF | 181.6 ± 16.5 (5) | 40.7 ± 3.9 (5) | 3.3 ± 0.5 (5) | 1136.3 ± 120 (5) | 0.22 ± 0.02 (5) | 99.3 ± 10.4 (5) |
| B2D + FF | 194.1 ± 26.7 (5) | 39.4 ± 3.8 (5) | 3.7 ± 0.5 (4) | 1142.6 ± 118 (5) | 0.13 ± 0.03 (5) | 76.6 ± 10.6 (5) |
| p | 0.70 | 0.82 | 0.82 | 0.84 | **0.04** | 0.16 |
