# Supplementary material for: Vitamin B2 enables regulation of fasting glucose availability
Source: eLife. 2023 Jul 7;12:e84077. doi: 10.7554/eLife.84077 (PMC10328530; doi:10.7554/eLife.84077)
Supplement: Figure 2—source data 3. — Data are mean ± SEM. The number of animals in each group is indicated in parentheses. Statistical significance (p) was determined by Mann–Whitney tests. [file elife-84077-fig2-data3.docx]

|  | **TGs (mg/g)** | | | **Cholesterol (mg/g)** | |
| --- | --- | --- | --- | --- | --- |
|  |  | **p** | |  | **p** |
| 99% Ctrl | 22.4 ± 2.1 (4) | | **0.05** | 1.1 ± 0.2 (4) | 0.26 |
| 99% B2D | 32.08 ± 3.45 (3) | |  | 0.8 ± 0.1 (4) |  |
|  |  |  | |  |  |
| Ctrl + FF | 8.3 ± 1.4 (4) | 0.24 | | 0.7 ± 0.1 (4) | **0.02** |
| B2D + FF | 11.8 ± 2.3 (4) |  | | 0.2 ± 0.1 (4) |  |
