# Supplementary figures and images for: Vitamin B2 enables regulation of fasting glucose availability
Source: eLife. 2023 Jul 7;12:e84077. doi: 10.7554/eLife.84077 (PMC10328530; doi:10.7554/eLife.84077)

Figure 5d

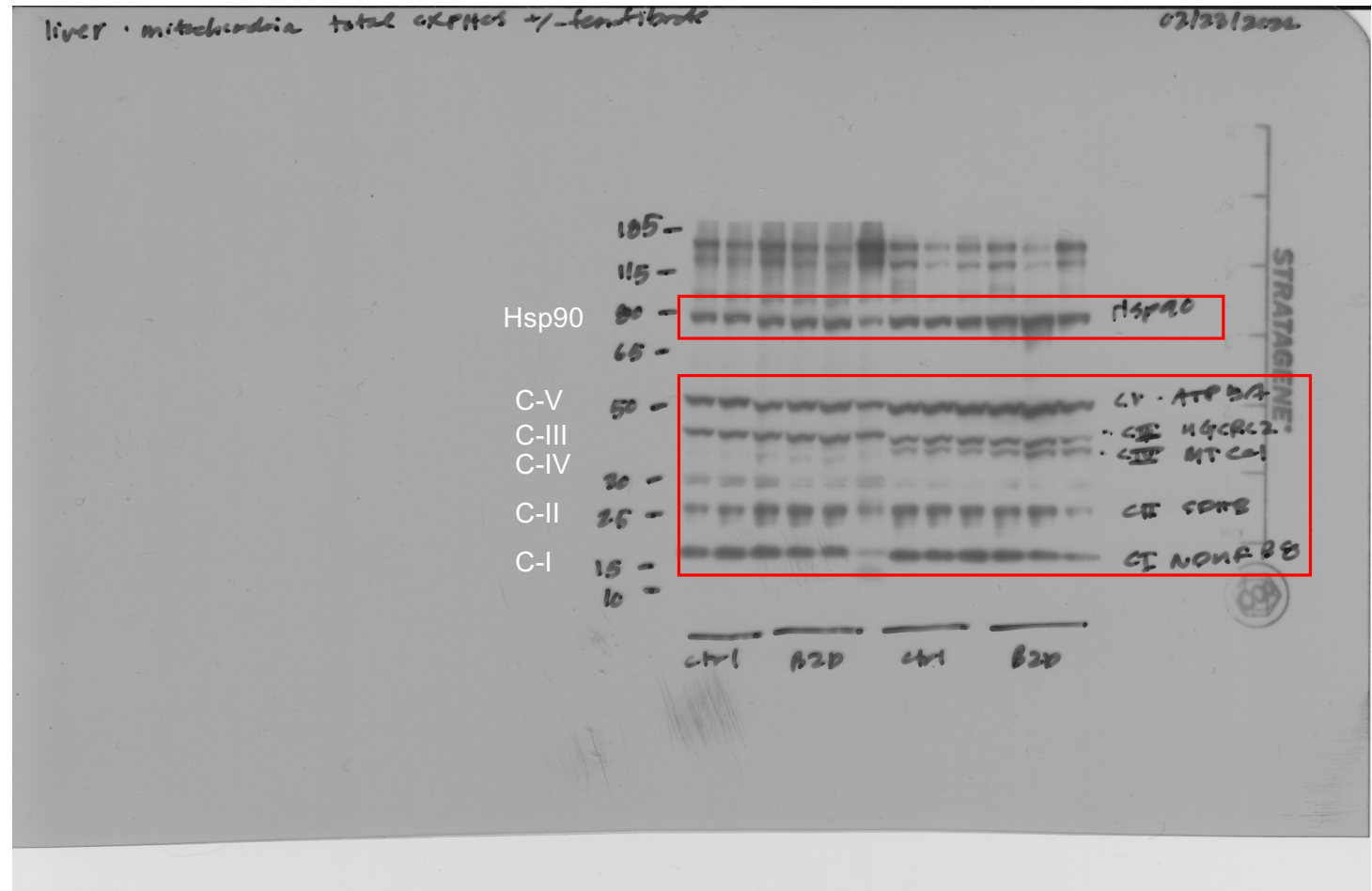

Supplement: Figure 5—source data 2. [file elife-84077-fig5-data2.zip › Figure 5D - source data file 1.pdf]

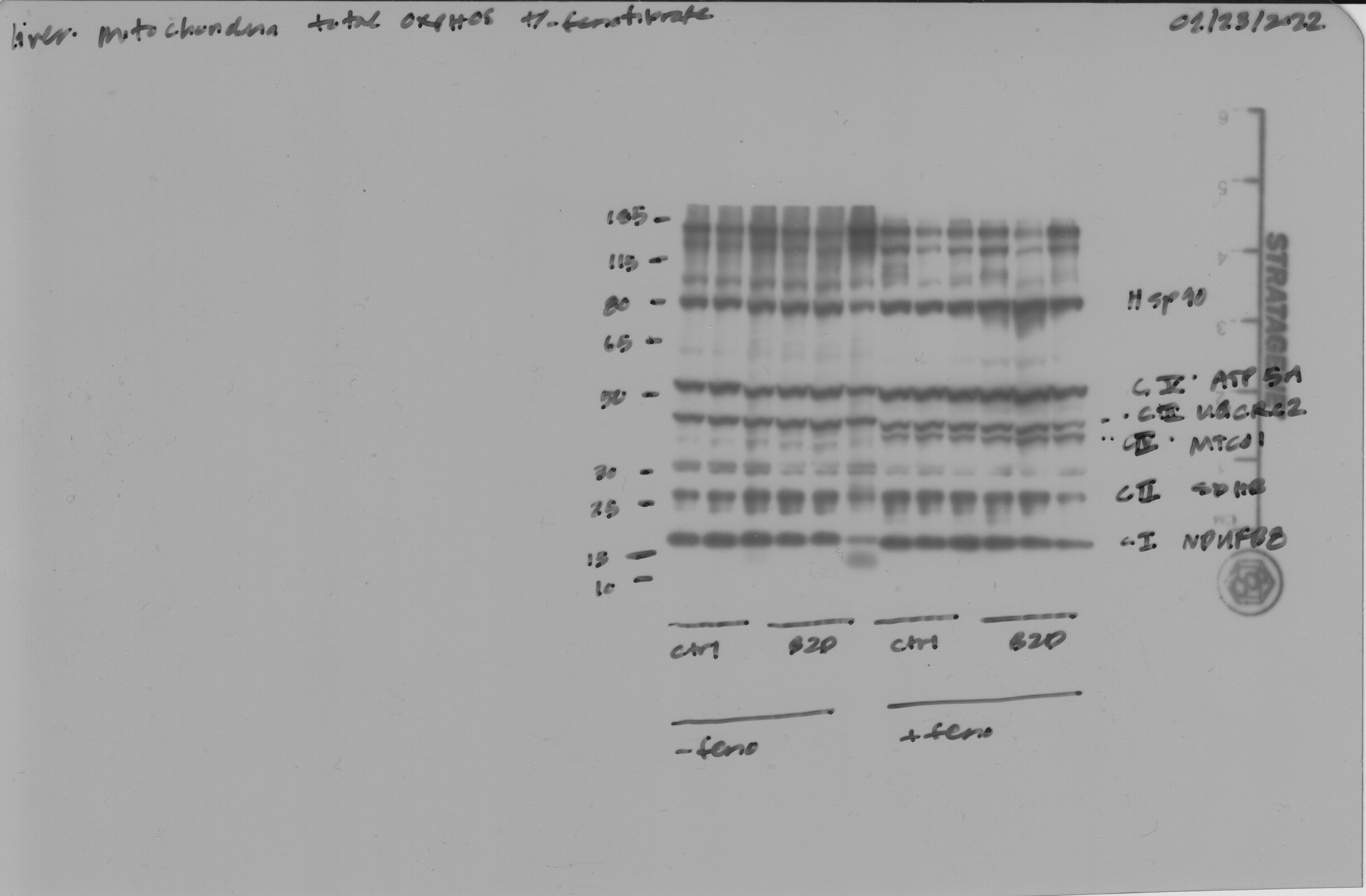

Supplement: Figure 5—source data 2. [file elife-84077-fig5-data2.zip › Figure5D_OXPHOS_HSP90_scan.tiff]

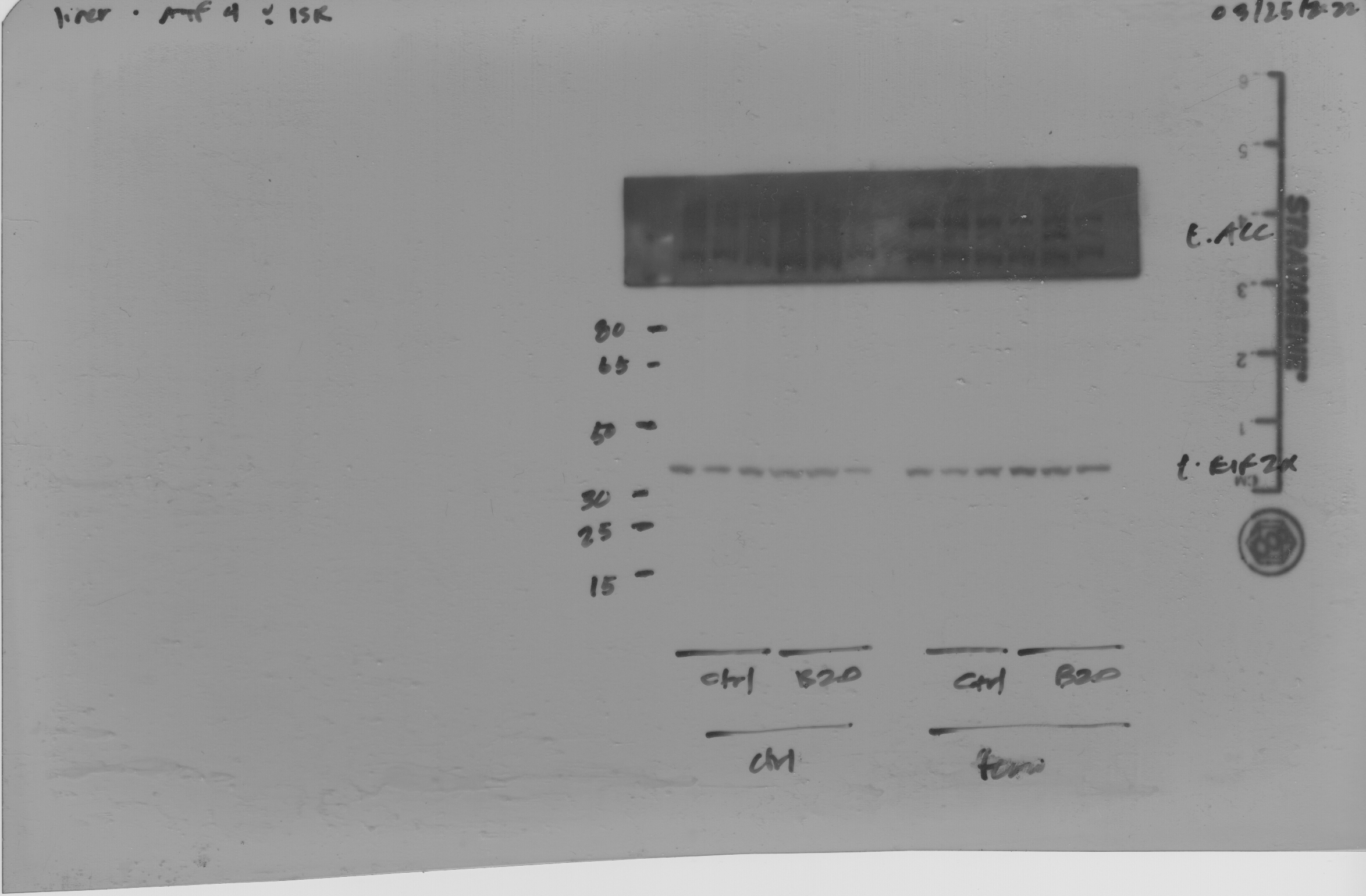

Supplement: Figure 6—source data 2. [file elife-84077-fig6-data2.zip › Figure6E_scan_tEIF2a.tif]

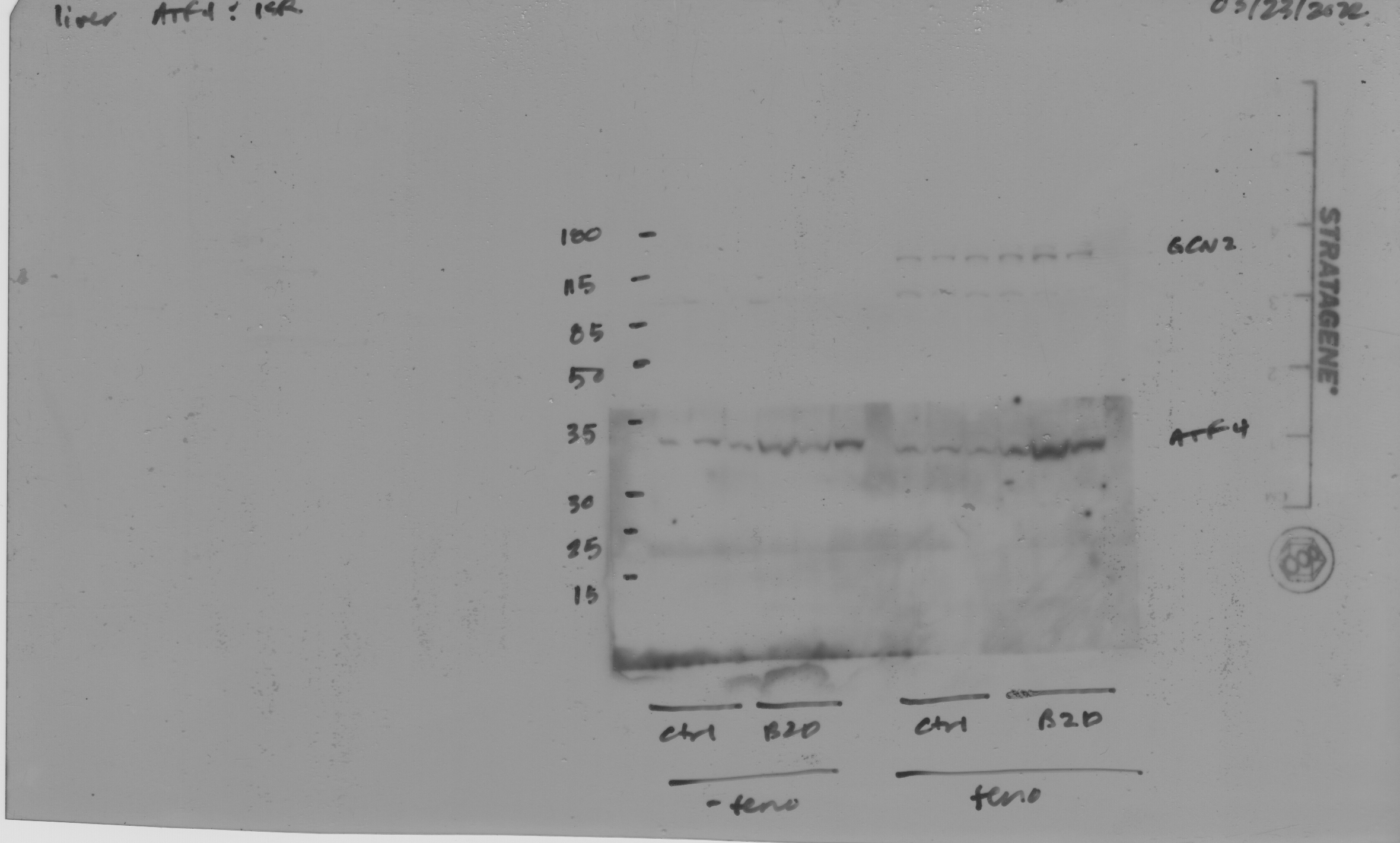

Supplement: Figure 6—source data 2. [file elife-84077-fig6-data2.zip › Figure6E_scan_GCN2_ATF4.tif]

Figure 6e

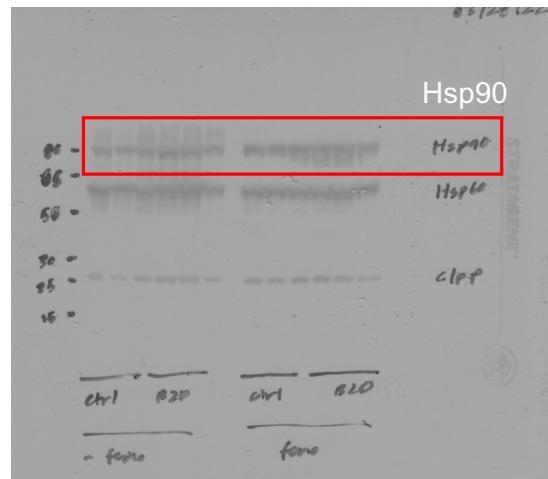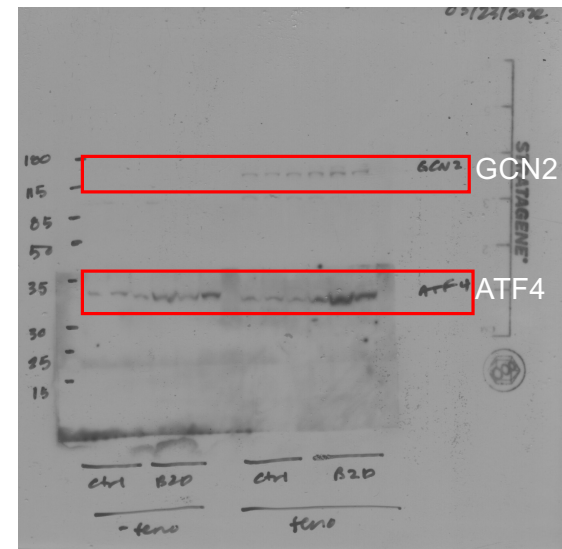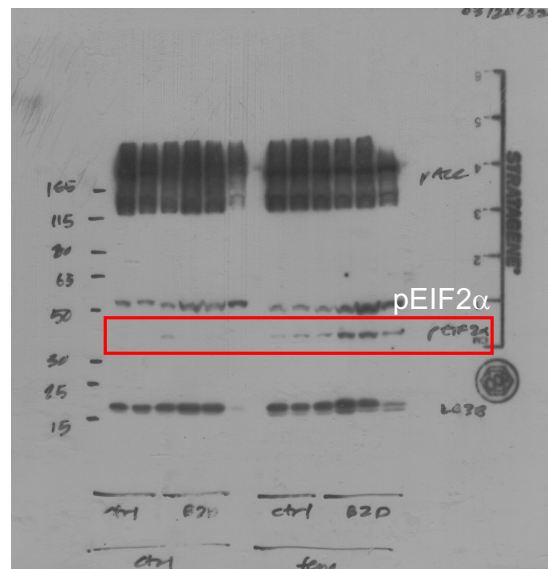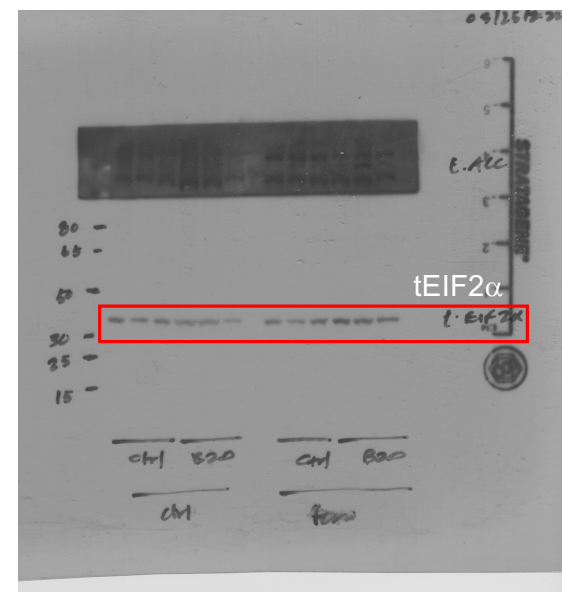

Supplement: Figure 6—source data 2. [file elife-84077-fig6-data2.zip › Figure_6E - source data 1.pdf]

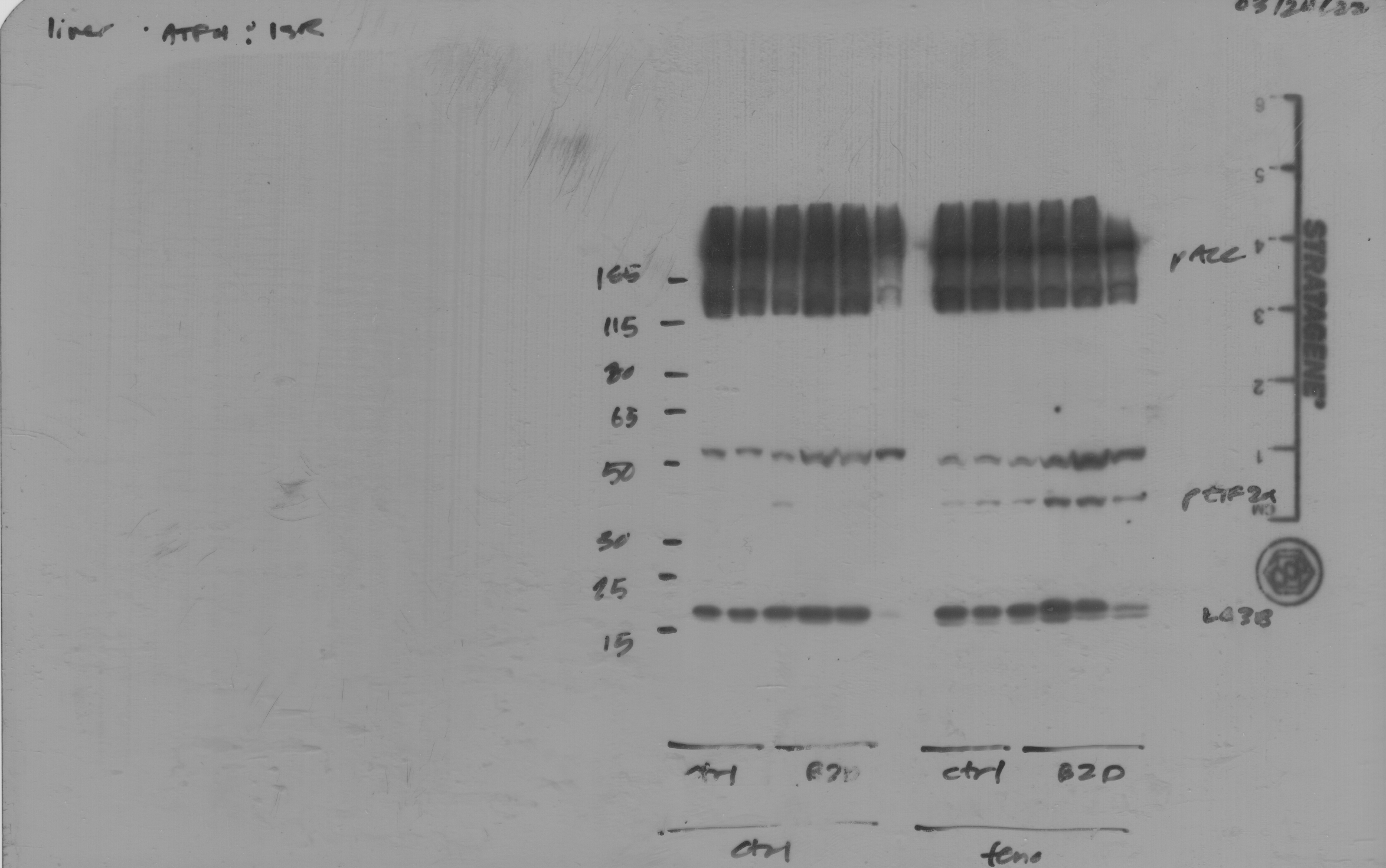

Supplement: Figure 6—source data 2. [file elife-84077-fig6-data2.zip › Figure6E_scan_pEIF2a.tif]

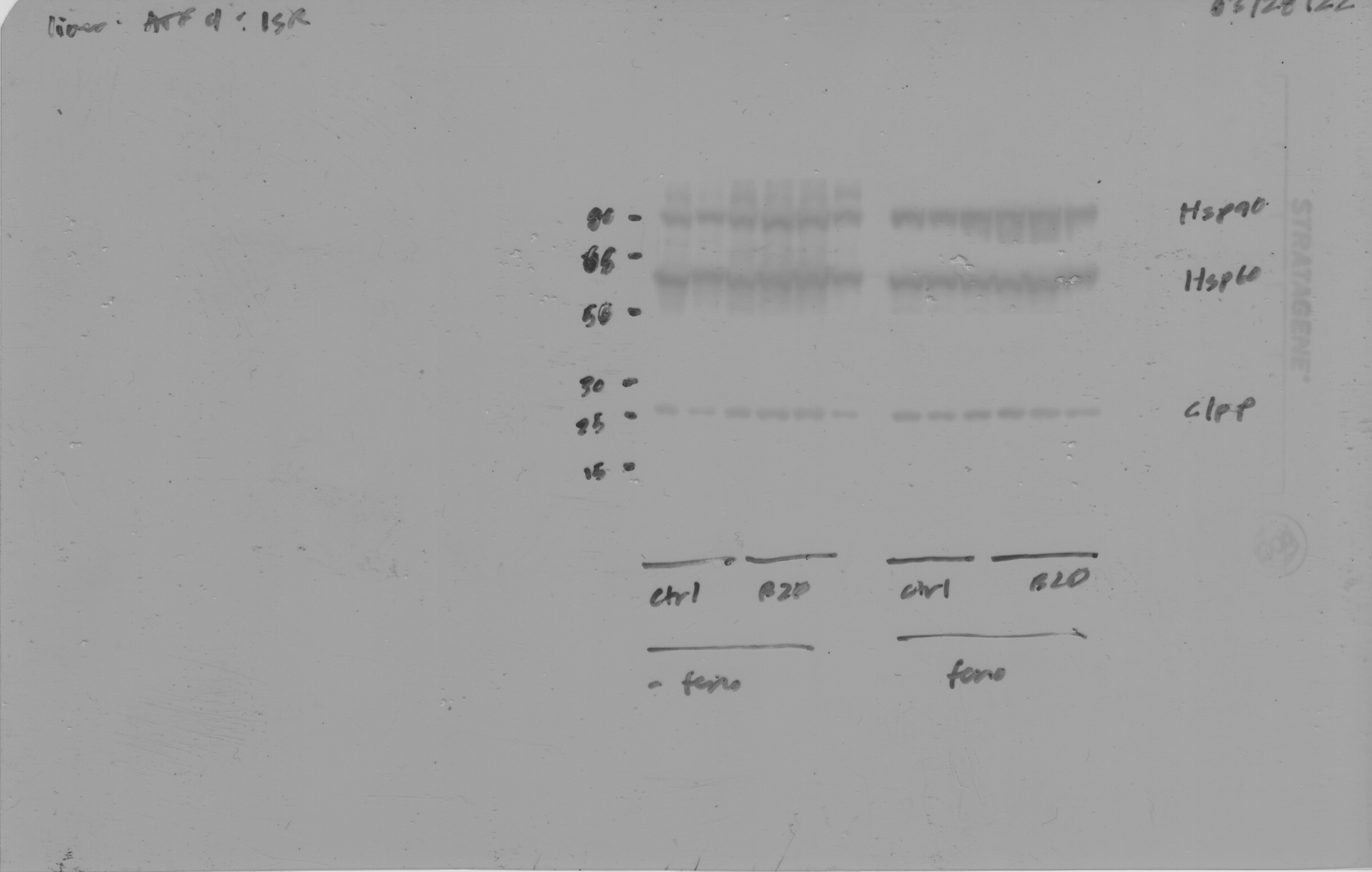

Supplement: Figure 6—source data 2. [file elife-84077-fig6-data2.zip › Figure6E_scan_Hsp90.tif]
