# Supplementary material for: Vitamin B2 enables regulation of fasting glucose availability
Source: eLife. 2023 Jul 7;12:e84077. doi: 10.7554/eLife.84077 (PMC10328530; doi:10.7554/eLife.84077)
Supplement: Supplementary file 1. [file elife-84077-supp1.zip › Supplemental_File_3.docx]

|  | **90% Ctrl** | **90% Deficient** |
| --- | --- | --- |
| Research Diets Name | AIN-93G Diet | AIN-93G Diet With No Added Riboflavin |
| Research Diets Number | D10012G | D12030102 |
| **Riboflavin Concentration** | **0.00698 g/kg** | **0.00098 g/kg** |
| **Formula** | **g/kg** | **g/kg** |
| Casein (0.00049g/kg riboflavin) | 200 | 200 |
| L-Cystine | 3 | 3 |
| Sucrose | 100 | 100 |
| Corn Starch | 397.486 | 397.486 |
| Maltodextrin 10 | 132 | 132 |
| Cellulose | 50 | 50 |
| Soybean Oil | 70 | 70 |
| t-butyrlhydroquinone | 0.014 | 0.014 |
| Choline Bitartrate | 2.5 | 2.5 |
| Mineral Mix S10022G | 35 | 35 |
| Vitamin Mix V10037 (0.006 g/kg Riboflavin) | 10 | 0 |
| Vitamin Mix V15920, No Added Riboflavin | 0 | 10 |
| **% kcal from** |  |  |
| Protein | 20.3 | 20.3 |
| Carbohydrates | 63.9 | 63.9 |
| Fat | 7 | 7 |
| Kcal/g | 4 | 4 |
