# Supplementary material for: Vitamin B2 enables regulation of fasting glucose availability
Source: eLife. 2023 Jul 7;12:e84077. doi: 10.7554/eLife.84077 (PMC10328530; doi:10.7554/eLife.84077)
Supplement: Supplementary file 2. [file elife-84077-supp2.zip › Supplemental_File_4.docx]

|  | **99% Ctrl** | **99% Deficient (B2D)** |
| --- | --- | --- |
| Research Diets Name | Modified AIN-93G Diet With L-Amino Acids | Modified AIN-93G Diet With L-Amino Acids and No Added Riboflavin |
| Research Diets Number | A18041301 | A19080901 |
| **Riboflavin Concentration** | **0.006 g/kg** | **0 g/kg** |
| **Formula** | **g/kg** | **g/kg** |
| L-Alanine | 5 | 5 |
| L-Arginine | 5.9 | 5.9 |
| L-Asparagine, monohydrate | 7 | 7 |
| L-Aspartic Acid | 5 | 5 |
| L-Cysteine | 4.2 | 4.2 |
| L-Glutamic Acid | 20.7 | 20.7 |
| L-Glutamine | 17.1 | 17.1 |
| Glycine | 3 | 3 |
| L-Histidine-HCl, monohydrate | 4.5 | 4.5 |
| L-Isoleucine | 7.5 | 7.5 |
| L-Leucine | 15.7 | 15.7 |
| L-Lysine, HCl | 13.1 | 13.1 |
| L-Methionine | 5 | 5 |
| L-Phenylalanine | 8.4 | 8.4 |
| L-Proline | 17.6 | 17.6 |
| L-Serine | 9.9 | 9.9 |
| L-Threonine | 7.1 | 7.1 |
| L-Tryptophan | 2.1 | 2.1 |
| L-Tyrosine | 9.1 | 9.1 |
| L-Valine | 9.2 | 9.2 |
| Sucrose | 107.0777 | 107.0777 |
| Corn Starch | 397.486 | 397.486 |
| Maltodextrin 10 | 132 | 132 |
| Cellulose | 50 | 50 |
| Soybean Oil | 70 | 70 |
| t-butyrlhydroquinone | 0.014 | 0.014 |
| Calcium Carbonate | 7.34 | 7.34 |
| Potassium Citrate 1, monohydrate | 2.4773 | 2.4773 |
| Potassium Phosphate, monobasic | 6.86 | 6.86 |
| Calcium Phosphate, dibasic | 7 | 7 |
| Sodium Chloride | 2.59 | 2.59 |
| Sodium Bicarbonate | 7.5 | 7.5 |
| Choline Bitartrate | 2.5 | 2.5 |
| Mineral Mix S10022C | 3.5 | 3.5 |
| Vitamin Mix V10037 (0.006 g/kg Riboflavin) | 10 | 0 |
| Vitamin Mix V15920, No added riboflavin | 0 | 10 |
| **% kcal from** |  |  |
| Protein | 18 | 18 |
| Carbohydrates | 65.9 | 65.9 |
| Fat | 7.1 | 7.1 |
| Kcal/g | 3.924 | 3.924 |
