# Supplementary material for: Vitamin B2 enables regulation of fasting glucose availability
Source: eLife. 2023 Jul 7;12:e84077. doi: 10.7554/eLife.84077 (PMC10328530; doi:10.7554/eLife.84077)
Supplement: Supplementary file 3. [file elife-84077-supp3.zip › Supplemental_File_5.docx]

| **Antibody** | **Company** | **Catalog Number** | **RRID** |
| --- | --- | --- | --- |
| Hsp90 | Cell Signaling | C45G5 | AB_2233307 |
| Gcn2 | Cell Signaling | 3302 | AB_2277617 |
| p-Eif2a | Cell Signaling | 9721 | AB_330951 |
| Eif2a | Santa Cruz Biotech | Sc133132 | AB_1562699 |
| Atf4 | Cell Signaling | 11815 | AB_2616025 |
| Total OXPHOS Rodent Cocktail | Abcam | Ab110413 | AB_2629281 |
| Rabbit HRP | Cell Signaling | 7074S | AB_2099233 |
| Mouse HRP | Cell Signaling | 7076P2 | AB_330924 |
